# The Psychological Impact of Hospital Admission on the Elderly: A Prospective Comparative Clinical Audit and Service Evaluation

**DOI:** 10.7759/cureus.117440

**Published:** 2026-10-04

**Authors:** Aqsa Sameen, Pirya Sarupani, Athina Angelopoulou

**Affiliations:** 1 Geriatrics, East Cheshire NHS Trust, Macclesfield, GBR

**Keywords:** corridor care, depression in medical disease, hospital admission, hospital isolation, reduced mobility, social impact, virtual ward, waiting times

## Abstract

Introduction

Hospital admission in older adults can be associated with functional decline, loss of independence, delirium, disruption of routine, and psychological deterioration. Virtual Wards, also described as Hospital-at-Home models, provide acute clinical care in a patient’s usual place of residence and may reduce some harms associated with inpatient admission. Although Virtual Wards are commonly evaluated in terms of bed capacity, admission avoidance, and clinical safety, their psychological impact remains less well explored. This audit compared depressive symptoms and self-rated mood in older adults managed in hospital with those treated through a Virtual Ward service.

Methods

A prospective comparative clinical audit and service evaluation was conducted within the Care of the Elderly directorate at East Cheshire NHS Trust. The audit included 100 patients aged ≥70 years: 50 hospital inpatients and 50 patients managed through Virtual Ward pathways. Eligible patients had received at least five days of inpatient or Virtual Ward care and had broadly comparable medical diagnoses. Patients with severe acute or life-threatening illness, those lacking capacity to participate, and those with communication barriers preventing completion of the assessment tools were excluded. Baseline age, sex, primary diagnosis, and Clinical Frailty Scale (CFS) characteristics were compared descriptively between cohorts. Depressive symptoms were assessed using the 15-item Geriatric Depression Scale (GDS-15), and self-rated mood was assessed using a five-point mood scale. Exploratory unadjusted between-group comparisons were performed for selected patient-level outcomes.

Results

Hospital inpatients demonstrated a greater depressive symptom burden than Virtual Ward patients. A GDS-15 score of ≥5 was recorded in 36 (72%) hospital inpatients compared with 15 (30%) Virtual Ward patients, corresponding to an unadjusted odds ratio of 6.00 (95% CI, 2.53-14.24; Fisher’s exact p<0.001). Low self-rated mood, defined as a score of 1 or 2, was reported by 19 (38%) hospital inpatients compared with three (6%) Virtual Ward patients, with an unadjusted odds ratio of 9.60 (95% CI, 2.62-35.21; Fisher’s exact p<0.001). Conversely, positive mood scores of 4 or 5 were reported by 32 (64%) Virtual Ward patients compared with 16 (32%) hospital inpatients. Baseline age and frailty distributions showed substantial descriptive overlap, although some variation in sex and primary diagnosis was present between cohorts.

Conclusion

Older adults receiving inpatient care demonstrated a greater burden of depressive symptoms and less positive self-rated mood than those managed through the Virtual Ward service. However, the non-randomised design, potential selection bias, and absence of adjustment for important baseline factors such as cognitive status, functional status, social support, and baseline psychological wellbeing mean that causation cannot be established. The findings should therefore be interpreted as an association between care setting and psychological wellbeing rather than evidence that Virtual Ward care itself improves mood. Further prospective evaluation using comprehensive baseline assessment, adjusted analyses, and post-discharge follow-up is required. Older adults who require hospital admission may also benefit from routine mood assessment and targeted measures to support psychological wellbeing.

## Introduction

Hospital admission is often necessary for the management of acute illness in older adults; however, for this population, the consequences of admission may extend beyond the primary medical diagnosis. Older patients are particularly vulnerable to hospital-associated decline because of frailty, multimorbidity, cognitive impairment, reduced physiological reserve, and dependence on familiar routines and social support [1,2]. In England, the scale of acute care demand remains substantial, with 27.4 million Accident and Emergency attendances reported in 2024-25, representing a 4.0% increase compared with the previous year [1]. Within this context, the care of older adults requires not only safe clinical treatment but also attention to function, independence, mood, and psychological wellbeing.

Hospitalisation in later life has been associated with loss of independence in activities of daily living, reduced mobility, delirium, and increased care needs after discharge [2]. These outcomes may occur even when the acute medical condition improves. The hospital environment can expose older adults to unfamiliar surroundings, sleep disturbance, noise, reduced privacy, disruption of routine, limited family contact, reduced autonomy, and anxiety about illness or dependency. These factors may contribute to emotional deterioration, low mood, and depressive symptoms. Depression among older medical inpatients is common and may be under-recognised in routine clinical practice, particularly where assessment is focused primarily on acute physical illness [3,4]. Psychological decline during admission may delay rehabilitation, reduce engagement with therapy, increase dependence, and complicate discharge planning.

Virtual Wards, also referred to as Hospital-at-Home models, have been developed to provide hospital-level care in a patient’s usual place of residence [5]. NHS England describes Virtual Wards as services that allow patients to receive the care they need safely and conveniently at home, either by preventing avoidable hospital admission or supporting earlier discharge from hospital [5]. The national Virtual Ward programme was introduced as part of a wider strategy to expand care outside hospital, improve patient flow, and deliver acute care closer to home. NHS England has set an ambition to scale Virtual Ward capacity towards 40-50 beds per 100,000 population, supported by service review, clinical audit, diagnostics, and technology-enabled care [6].

Assessment of depressive symptoms in older adults can be supported by the 15-item Geriatric Depression Scale (GDS-15), a short-form screening tool developed for use in older populations [7]. The GDS-15 has also been evaluated in very old people with and without cognitive impairment [8]. More recent evidence demonstrates a substantial burden of depression among older adults, reinforcing the importance of recognising psychological symptoms alongside physical illness in later life [9]. Recent evidence syntheses have also examined Virtual Ward and Hospital-at-Home models, supporting an expanding evidence base for the delivery of inpatient-level care at home while highlighting the need for continued evaluation of these services [10,11]. Within East Cheshire NHS Trust, the Virtual Ward service began in January 2023 and supports patients through daily clinical reviews, remote monitoring, and home visits.

For older adults, the potential benefits of Virtual Ward care may extend beyond admission avoidance and bed capacity. Receiving acute care at home may preserve familiar surroundings, sleep patterns, mobility, nutrition, family interaction, personal routine, and a sense of autonomy. These factors may be clinically relevant to recovery, rehabilitation potential, and overall quality of life in older people. Although Virtual Wards are increasingly evaluated through operational and clinical outcomes, including escalation rates, length of stay, admission avoidance, and service capacity, comparatively less attention has been given to whether the care environment itself is associated with mood and depressive symptoms in older adults.

This clinical audit was designed to explore this gap by comparing depressive symptoms and self-rated mood between older adults managed as hospital inpatients and those cared for through the Virtual Ward service. The audit was undertaken within the Care of the Elderly directorate at East Cheshire NHS Trust and was registered and approved by the Clinical Effectiveness Team as Audit COTE2501. It compared 50 inpatients from medical wards with 50 Virtual Ward patients from frailty, respiratory, and general medicine pathways. Depressive symptoms were assessed using the 15-item Geriatric Depression Scale, and self-rated mood was assessed using a five-point mood scale. The aim was to assess whether the setting of care was associated with differences in depressive symptom burden and mood, and to inform future service development, including earlier Virtual Ward referral, routine mood screening, and targeted emotional wellbeing interventions for older inpatients.

## Materials and methods

This was a prospective comparative clinical audit and service evaluation conducted at Macclesfield District General Hospital, East Cheshire NHS Trust, within the Care of the Elderly directorate. The audit compared older adults receiving care on hospital inpatient wards with those managed through the Trust’s Virtual Ward service.

The audit included 100 patients aged 70 years and above. Fifty patients were recruited from medical inpatient wards, and 50 were recruited from the Virtual Ward service, including frailty, respiratory, and general medicine pathways. Patients were eligible for inclusion if they were aged ≥70 years, had received at least five days of inpatient or Virtual Ward care, and had broadly comparable medical diagnoses. Patients were excluded if they had severe acute or life-threatening illness, lacked capacity to participate, or had communication barriers that prevented completion of the assessment tools. Participants were medically optimised at the time of assessment. No participants were recruited from nursing or residential care homes because the Virtual Ward service was not available in these settings during the audit period.

Baseline demographic and clinical characteristics recorded for descriptive comparison included age group, sex, primary diagnosis, Clinical Frailty Scale (CFS), and nursing or residential care-home status. Detailed baseline cognitive status, living arrangements, and measures of social support were not systematically collected and therefore could not be compared between the two cohorts.

Depressive symptoms were assessed using the 15-item Geriatric Depression Scale (GDS-15), a validated short-form screening tool developed for use in older adults (Table 1) [7]. The GDS-15 uses a yes/no response format and focuses primarily on psychological and cognitive symptoms of depression, making it suitable for older adults in whom somatic symptoms may overlap with physical illness [7,8]. Self-rated emotional wellbeing was assessed using a five-point mood scale ranging from 1 (very low mood) to 5 (very positive mood) (Figure 1). This patient-facing measure was used alongside the GDS-15 to capture subjective emotional wellbeing. Hospital inpatients completed the assessments at the bedside, whereas Virtual Ward patients completed structured assessments during telephone or home-based reviews. All responses were anonymised before analysis.

**Table 1:** Geriatric Depression Scale (GDS-15) Items and Scoring The GDS-15 is a 15-item yes/no screening tool for depressive symptoms in older adults. Each response is scored as 0 or 1, giving a total score ranging from 0 to 15. A score of 5 or above may indicate depressive symptoms and should prompt further clinical assessment. Adapted from Sheikh and Yesavage [7].

Item	Question	Yes score	No score
1	Are you basically satisfied with your life?	0	1
2	Have you dropped many of your activities and interests?	1	0
3	Do you feel that your life is empty?	1	0
4	Do you often get bored?	1	0
5	Are you in good spirits most of the time?	0	1
6	Are you afraid that something bad is going to happen to you?	1	0
7	Do you feel happy most of the time?	0	1
8	Do you often feel helpless?	1	0
9	Do you prefer to stay at home, rather than going out and doing new things?	1	0
10	Do you feel you have more problems with memory than most?	1	0
11	Do you think it is wonderful to be alive now?	0	1
12	Do you feel pretty worthless the way you are now?	1	0
13	Do you feel full of energy?	0	1
14	Do you feel that your situation is hopeless?	1	0
15	Do you think that most people are better off than you are?	1	0

**Figure 1:**
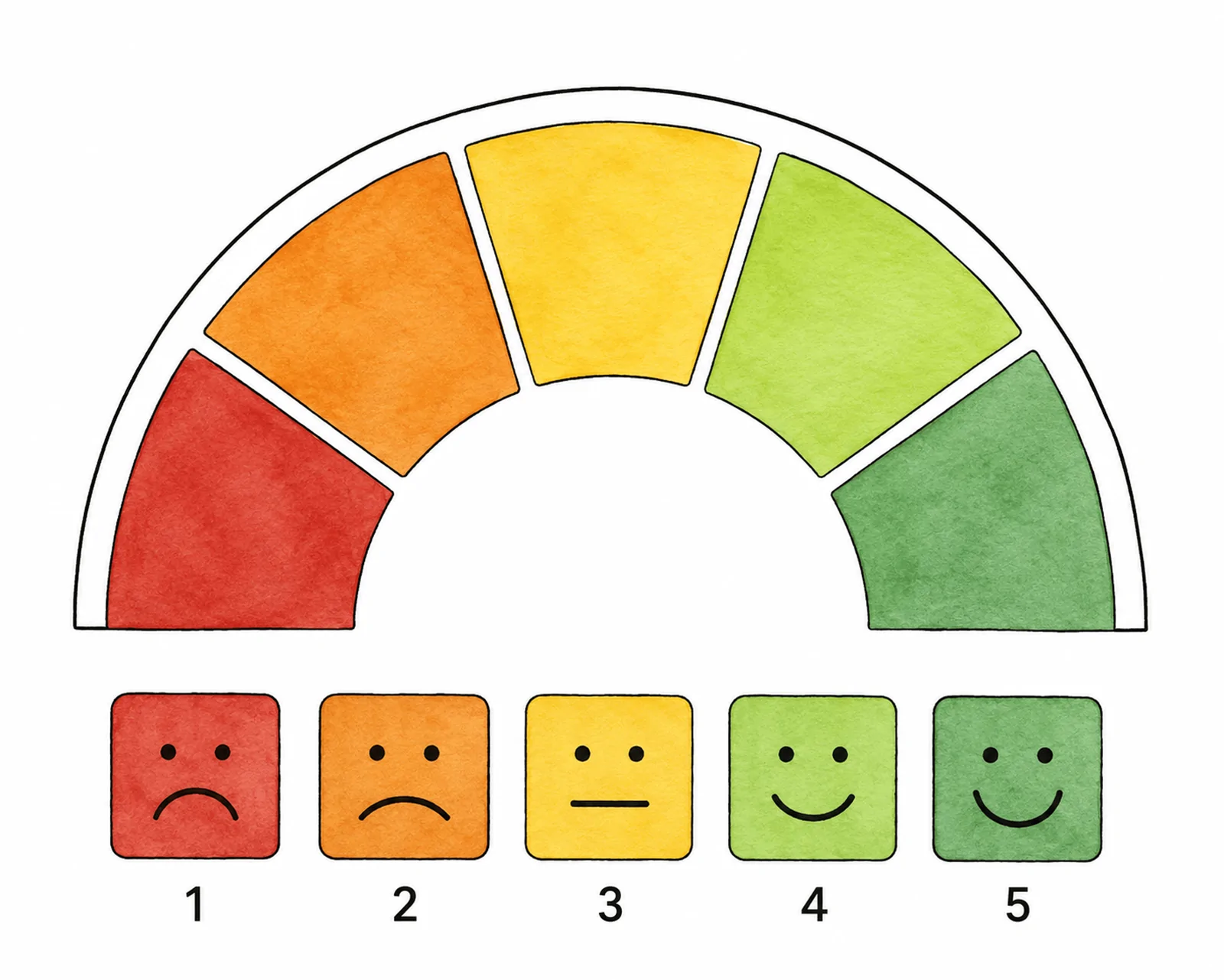
Five-Point Mood Scale for Self-Rated Emotional Wellbeing A five-point mood scale was used as a simple patient-facing aid to assess self-rated emotional wellbeing. Scores ranged from 1 to 5, where 1 represented very low mood and 5 represented very positive mood. The scale was used alongside the Geriatric Depression Scale-15 to support discussion of subjective mood during inpatient and Virtual Ward care. Source: Authors’ own illustration created in Microsoft PowerPoint.

The primary outcome was the difference in depressive symptom burden between the inpatient and Virtual Ward cohorts, measured by the proportion of depression-scored responses across the GDS-15 items. Secondary outcomes included differences in self-rated mood and patterns of responses across individual GDS-15 items, including reduced activities and interests, feelings of emptiness or worthlessness, low energy, and hopelessness.

The analysis was primarily descriptive. As this project was conducted as a prospective comparative clinical audit and service evaluation, its primary purpose was to evaluate existing care pathways and identify potential opportunities for service improvement rather than to test an experimental intervention. Baseline demographic and clinical characteristics and other categorical data were summarised as numbers and percentages. Differences between the inpatient and Virtual Ward cohorts were described using absolute differences where appropriate.

Exploratory inferential analyses were also performed for selected patient-level outcomes. A GDS-15 score of ≥5 was used to identify participants with depressive symptoms warranting further clinical assessment, in accordance with the established interpretation of the GDS-15. Low self-rated mood was defined as a score of 1 or 2 on the five-point mood scale. Fisher’s exact test was used to compare these binary outcomes between the two cohorts. Unadjusted odds ratios, absolute differences in proportions, and corresponding 95% confidence intervals were calculated where appropriate. These analyses were exploratory and were not adjusted for potential baseline confounding variables; therefore, they should not be interpreted as demonstrating a causal effect of care setting.

This project was conducted as a registered clinical audit and service evaluation rather than as an interventional research study. Formal written informed consent for research participation was not required. All participants had capacity to participate, and patients provided verbal consent before completing the mood and wellbeing assessments. Only anonymised data were used for service evaluation and quality improvement. No identifiable patient information was included in the analysis or manuscript.

## Results

A total of 100 patients were included in the final analysis, comprising 50 hospital inpatients and 50 patients managed through the Virtual Ward service. All patients were aged 70 years or older and had received at least five days of care within their respective pathways.

Depressive symptoms were assessed using the GDS-15, with item scoring presented in Table 1. Hospital inpatients demonstrated a higher overall burden of depressive symptoms than patients managed through the Virtual Ward. The average proportion of depression-scored responses across the 15 GDS-15 items was 47% in the inpatient cohort and 18% in the Virtual Ward cohort, representing an absolute difference of 29 percentage points.

Analysis of individual GDS-15 items showed higher proportions of depression-scored responses among hospital inpatients across all 15 items. The largest differences were observed for reduced activities and interests, feelings of emptiness, low spirits, worthlessness, and hopelessness. Across individual items, depression-scored responses ranged from 6 (12%) to 43 (86%) among hospital inpatients and from 3 (6%) to 17 (34%) among Virtual Ward patients (Figure 2).

**Figure 2:**
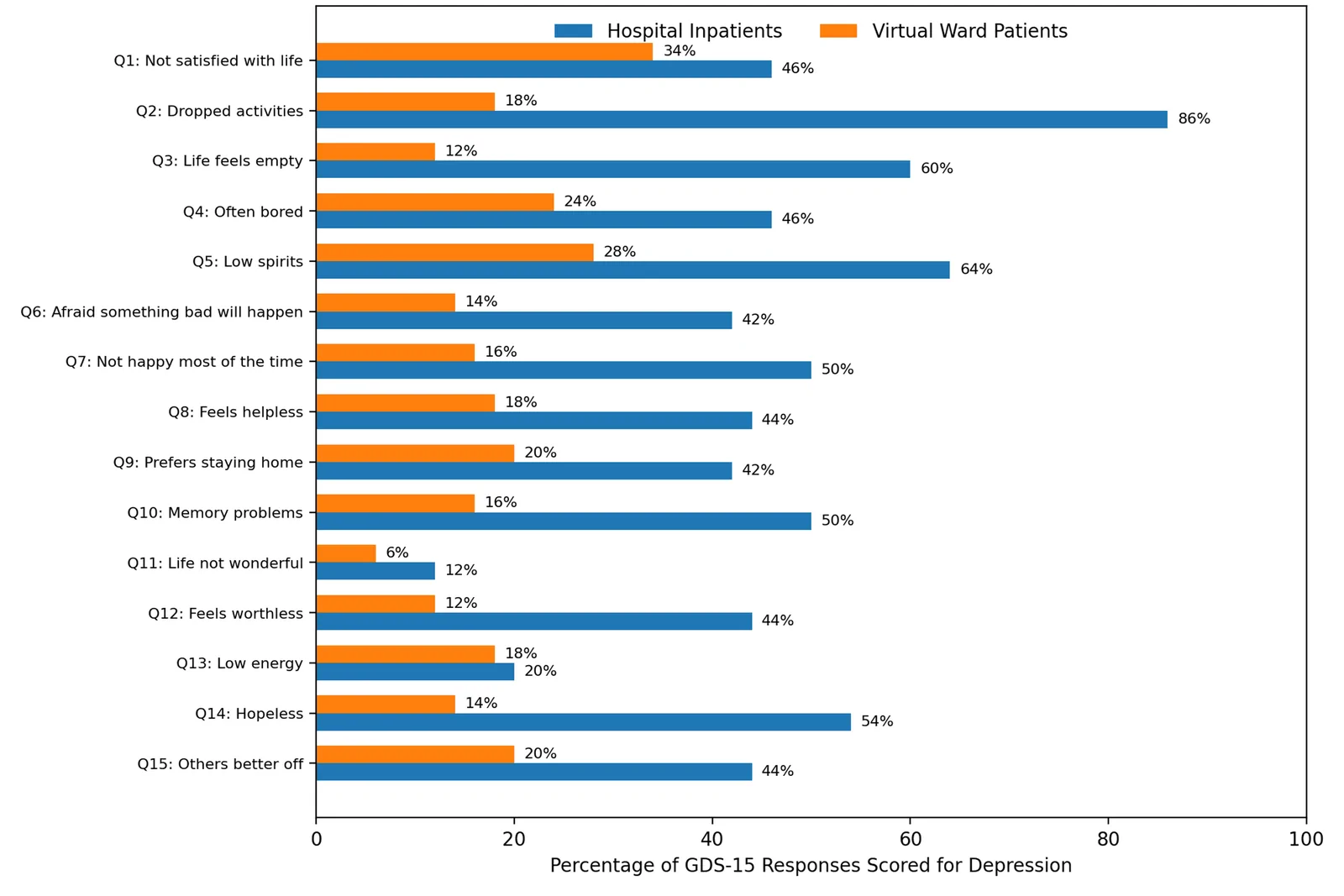
GDS-15 Depression-Scored Responses by Cohort The chart shows the proportion of patients with a depression-scored response for each of the 15 GDS-15 items in the hospital inpatient and Virtual Ward cohorts (N=50 per cohort), according to the scoring direction of each question. Across individual items, depression-scored responses ranged from 6 (12%) to 43 (86%) among hospital inpatients and from 3 (6%) to 17 (34%) among Virtual Ward patients. Hospital inpatients had a higher proportion of depression-scored responses across all 15 GDS-15 items. GDS-15: 15-Item Geriatric Depression Scale

The distribution of GDS-15 score categories differed between the two cohorts (Figure 3). Among hospital inpatients, 14 (28%) were in the normal category, 23 (46%) were in the mild category, nine (18%) were in the moderate category, and four (8%) were in the severe category. Among Virtual Ward patients, 35 (70%) were in the normal category, 10 (20%) were in the mild category, three (6%) were in the moderate category, and two (4%) were in the severe category. Using a GDS-15 score of ≥5 as the threshold indicating depressive symptoms warranting further clinical assessment, 36 (72%) hospital inpatients met this threshold compared with 15 (30%) Virtual Ward patients. This represented an absolute difference of 42 percentage points (95% CI: 22.6-57.2), with an unadjusted odds ratio of 6.00 (95% CI: 2.53-14.24; Fisher’s exact p<0.001). These findings indicate a higher depressive symptom burden among hospital inpatients; however, this comparison was exploratory and unadjusted.

**Figure 3:**
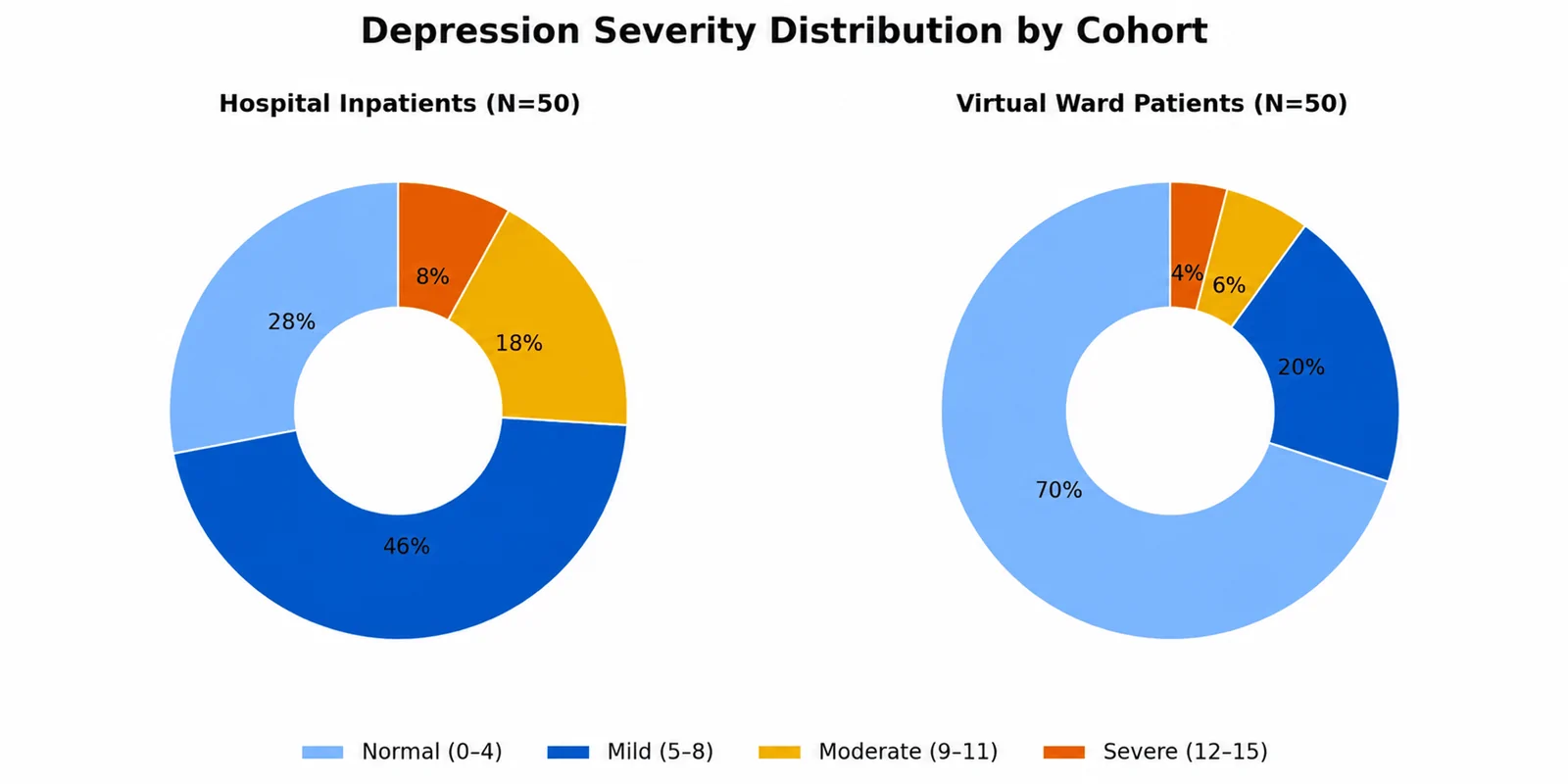
Depression Severity Distribution by Cohort GDS-15 total scores were grouped as normal (0–4), mild (5–8), moderate (9–11), and severe (12–15). Among hospital inpatients, 14 (28%) were in the normal category, 23 (46%) mild, nine (18%) moderate, and four (8%) severe. Among Virtual Ward patients, 35 (70%) were in the normal category, 10 (20%) mild, three (6%) moderate, and two (4%) severe. These categories describe depressive symptom burden on the GDS-15 and do not constitute a clinical diagnosis of depression. GDS-15: 15-item Geriatric Depression Scale

Self-rated mood followed a similar pattern (Figure 4). Using the five-point mood scale, 19 (38%) hospital inpatients rated their mood in the lowest two categories, corresponding to scores of 1 or 2, compared with 3 (6%) Virtual Ward patients. This represented an absolute difference of 32 percentage points (95% CI: 16.1-46.4), with an unadjusted odds ratio of 9.60 (95% CI: 2.62-35.21; Fisher’s exact p<0.001). Conversely, 32 (64%) Virtual Ward patients rated their mood in the highest two categories, corresponding to scores of 4 or 5, compared with 16 (32%) hospital inpatients. The mood distribution therefore shifted toward higher scores in the Virtual Ward cohort and lower scores in the hospital inpatient cohort. These comparisons were exploratory and were not adjusted for potential baseline confounders. 

**Figure 4:**
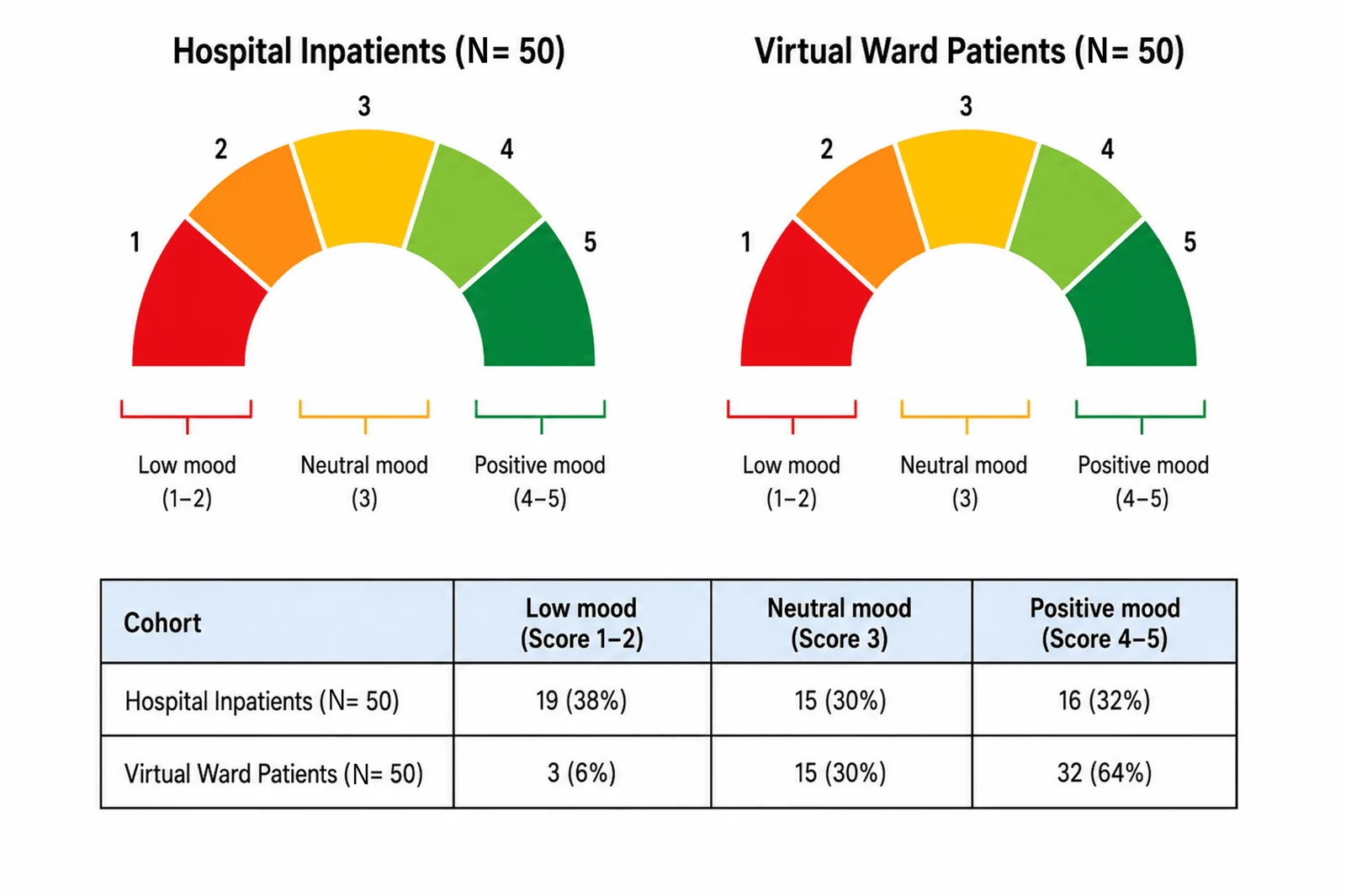
Five-Point Mood Scale Distribution by Cohort Distribution of five-point mood scale categories among hospital inpatients and Virtual Ward patients. Low mood represents scores of 1–2, neutral mood represents a score of 3, and positive mood represents scores of 4–5. Data are presented as N (%). Source: Authors’ own illustration created in Microsoft PowerPoint.

Baseline demographic and clinical characteristics of the hospital ward and Virtual Ward cohorts are summarised in Table 2.

**Table 2:** Baseline Demographic and Clinical Characteristics of the Hospital Ward and Virtual Ward Cohorts Data are presented as N (%). CFS: Clinical Frailty Scale; LRTI: lower respiratory tract infection. All participants had capacity to participate and were medically optimised at the time of assessment. Patients with severe acute or life-threatening illness were excluded. No nursing or residential care-home residents were included because the Virtual Ward service was not available in these settings during the audit period. Detailed baseline cognitive status, living arrangements, and measures of social support were not systematically collected and therefore could not be compared between cohorts.

Characteristic	Hospital Ward (N=50)	Virtual Ward (N=50)
Age 70–79 years, N (%)	18 (36%)	16 (32%)
Age 80–89 years, N (%)	32 (64%)	34 (68%)
Female, N (%)	35 (70%)	28 (56%)
Male, N (%)	15 (30%)	22 (44%)
CFS 1–3, N (%)	23 (46%)	24 (48%)
CFS 4, N (%)	23 (46%)	19 (38%)
CFS 5–6, N (%)	4 (8%)	7 (14%)
Urinary tract infection, N (%)	19 (38%)	23 (46%)
LRTI/pneumonia, N (%)	14 (28%)	18 (36%)
Heart failure, N (%)	9 (18%)	5 (10%)
Acute kidney injury, N (%)	8 (16%)	4 (8%)
Nursing/residential care-home resident, N (%)	0 (0%)	0 (0%)

## Discussion

This audit identified differences in GDS-15 depression-scored responses and self-rated mood between older adults managed as hospital inpatients and those cared for through a Virtual Ward model. Hospital inpatients had a higher average proportion of depression-scored responses across the GDS-15 items, while a greater proportion were represented in the mild, moderate, and severe GDS-15 score categories (Figures 2, 3). Self-rated mood showed a similar pattern, with hospital inpatients more likely to report low mood and Virtual Ward patients more likely to report positive mood on the five-point mood scale (Figure 4). These findings suggest an association between care setting and psychological wellbeing in older adults rather than establishing a causal effect of the care environment.

The findings are consistent with previous evidence showing that hospital admission in older adults can be associated with functional decline, loss of independence, and increased vulnerability after discharge [2]. Although inpatient care remains essential for many older patients, the hospital environment can be challenging because of unfamiliar surroundings, disrupted sleep, reduced mobility, noise, loss of routine, limited privacy, and separation from family or carers. These factors may contribute to emotional distress and depressive symptoms, particularly among patients with frailty, multimorbidity, cognitive vulnerability, or reduced physiological reserve [2].

Depressive symptoms and low mood in older medical inpatients are clinically important because they may reduce engagement with rehabilitation, mobility, nutrition, treatment decisions, and discharge planning. Previous literature has shown that depression in older medical inpatients may be under-recognised in routine clinical practice, particularly when assessment is focused primarily on physical illness [3,4]. More recent systematic review evidence also demonstrates a substantial burden of depression among older adults, reinforcing the importance of recognising and addressing psychological symptoms in later life [9]. In this audit, notable differences between the groups were observed in GDS-15 items relating to reduced activities and interests, feelings of emptiness, low spirits, worthlessness, and hopelessness (Figure 2). These symptoms may affect recovery, confidence, and participation in rehabilitation even after the acute medical condition has improved.

The lower burden of depression-scored responses observed in the Virtual Ward cohort may reflect several potentially protective features of home-based care. Virtual Wards allow selected patients to receive hospital-level care in their usual place of residence, supported by clinical review, monitoring, diagnostics, and escalation pathways [5,6]. For older adults, this model may help preserve familiar surroundings, family contact, sleep patterns, personal routines, independence, and autonomy. These factors may support emotional stability during illness. Recent evidence syntheses have examined Virtual Ward and Hospital-at-Home models in older people and inpatient-level home care. These reviews indicate a more established evidence base for Hospital-at-Home care while emphasising the need for continued rigorous evaluation of newer Virtual Ward models [10,11]. However, the observed differences in the present audit cannot be attributed solely to the care environment because the two groups were not randomly allocated and may have differed in important baseline characteristics.

The findings suggest that psychological wellbeing and patient experience should be considered alongside operational and clinical outcomes when evaluating Virtual Ward and hospital-based care models for older adults [5,6]. A recent systematic review of Hospital-at-Home care for older people highlighted the importance of familiar surroundings, person-centred care, shared decision-making, and support for patients and carers, while also identifying practical challenges in delivering home-based care [12]. Where clinically appropriate, Virtual Ward pathways may therefore offer advantages by allowing suitable patients to remain in a familiar environment. Early supported discharge is also relevant to home-based models of acute care; systematic review evidence in older medical patients suggests that it can reduce hospital length of stay, although evidence for improvement in other outcomes remains less conclusive [13]. For patients who require inpatient care, routine mood screening and targeted wellbeing interventions may help identify and support those at risk of psychological deterioration, particularly given the recognised under-detection of depression in older medical inpatients [3,4].

Several important limitations should be acknowledged, particularly the potential for selection bias and residual confounding. This was a non-randomised clinical audit, and patients were not randomly allocated to hospital inpatient or Virtual Ward care. All participants were aged 70 years or older, had capacity to participate in the assessment, and were medically optimised at the time of assessment. Patients with severe acute or life-threatening medical conditions were excluded in accordance with the eligibility criteria. No participants were recruited from nursing or residential care homes because the Virtual Ward service was not available in these settings during the audit period. These eligibility criteria reduced some obvious differences in clinical status but do not establish that the two cohorts were equivalent at baseline.

Baseline demographic and clinical characteristics were compared descriptively between the cohorts (Table 2). Among hospital inpatients, 18 (36%) were aged 70-79 years and 32 (64%) were aged 80-89 years, compared with 16 (32%) and 34 (68%), respectively, in the Virtual Ward cohort. Sex distribution showed some variation, with 35 (70%) women in the hospital cohort compared with 28 (56%) in the Virtual Ward cohort. Frailty distributions also showed substantial overlap: CFS scores of 1-3 were present in 23 (46%) hospital inpatients and 24 (48%) Virtual Ward patients, CFS 4 was recorded in 23 (46%) and 19 (38%), respectively, and CFS 5-6 in 4 (8%) and 7 (14%), respectively. Primary diagnoses included urinary tract infection, lower respiratory tract infection/pneumonia, heart failure, and acute kidney injury, with some variation in their distribution between cohorts. These descriptive comparisons provide useful information regarding baseline characteristics but should not be interpreted as demonstrating equivalence or successful matching between the groups.

Patients accepted onto the Virtual Ward had to be considered clinically suitable for management in their usual place of residence. This selection process means that patients managed through the Virtual Ward were likely to have been clinically more stable than those requiring continued inpatient care, introducing an important potential source of selection bias. Consequently, even though participants were medically optimised and those with severe acute or life-threatening illness were excluded, Virtual Ward patients may still have differed from hospital inpatients in ways that were not captured by the available baseline data. In particular, differences in functional independence, multimorbidity, cognitive status, baseline psychological wellbeing, living arrangements, and availability of family or social support may independently have influenced depressive symptoms and self-rated mood. These factors therefore remain potential sources of residual confounding.

Detailed baseline cognitive status, living arrangements, and measures of social support were not systematically collected and could not be formally compared or adjusted for in the analysis. Baseline mood before entry into the respective care pathways was also unavailable. Consequently, despite the additional baseline comparisons presented in Table 2, residual confounding and selection bias remain possible. The observed differences in depressive symptoms and self-rated mood should therefore be interpreted as associations between care setting and psychological outcomes rather than as evidence that Virtual Ward care itself caused better psychological outcomes.

Although age, sex, and CFS data were available, multivariable adjustment or matching was not undertaken because the sample size was modest and several important potential confounders, including detailed functional status, cognitive status, baseline mood, illness severity, living arrangements, and social support, were not systematically collected. Adjustment using only the available variables would therefore have left substantial residual confounding. The analytical approach also represents a limitation. This project was undertaken as a clinical audit and service evaluation, and the analysis was primarily descriptive rather than designed to test a causal hypothesis. Limited exploratory inferential analyses were performed for selected patient-level outcomes to quantify the observed between-group differences. These analyses were unadjusted and did not account for potentially important baseline confounders. Therefore, although the reported effect estimates, confidence intervals, and p-values provide additional information regarding the magnitude and statistical uncertainty of the observed differences, they should not be interpreted as demonstrating a causal effect of care setting. The manuscript reports these analyses as exploratory and unadjusted.

The sample size was modest, with 50 patients in each cohort, and the findings reflect the experience of a single NHS Trust, which may limit generalisability. The exclusion of nursing and residential care-home residents also means that the findings may not be generalisable to older adults living in institutional care settings. The exclusion of patients who lacked capacity or had communication barriers may further limit the generalisability of the findings to older adults with cognitive or communication impairment. Follow-up data were not collected to determine whether the observed differences in mood and depressive symptoms persisted following discharge or completion of Virtual Ward care. The assessment methods also differed between settings, with hospital inpatients assessed at the bedside and Virtual Ward patients assessed by telephone or during home-based reviews, which may have influenced how participants responded.

Despite these limitations, the audit identifies an important aspect of care for older adults that warrants further evaluation. The observed association between care setting and psychological wellbeing supports further prospective investigation rather than establishing the superiority of one model of care. For hospital inpatients, practical interventions such as routine mood screening, clear communication of care plans, promotion of mobility and sleep hygiene, family involvement, meaningful activity, and consistent staff engagement may help support psychological wellbeing during admission.

Future work should prospectively collect and compare comprehensive baseline characteristics, including detailed functional status, cognitive status, baseline mood, multimorbidity, illness severity, living arrangements, and social support. Larger studies should also consider appropriate multivariable adjustment, subgroup analyses where feasible, and post-discharge follow-up. Such studies would help determine whether the differences observed in this audit persist after accounting for baseline differences between patients and whether the care environment itself has an independent association with psychological outcomes.

## Conclusions

Older adults receiving inpatient care demonstrated a greater burden of depressive symptoms and less positive self-rated mood than those managed through the Virtual Ward service. Although baseline age, sex, frailty, and primary diagnosis were compared descriptively, the non-randomised design and lack of adjustment for other potentially important confounders mean that causation cannot be established. The findings should therefore be interpreted as an association between care setting and psychological wellbeing rather than as evidence that Virtual Ward care itself improves mood.

For clinically suitable patients, Virtual Ward care may provide an opportunity to receive acute care while remaining in a familiar environment. Older adults who require hospital admission may benefit from routine mood assessment and targeted measures supporting sleep, mobility, communication, meaningful activity, and family involvement. Further prospective evaluation with comprehensive baseline assessment, adjusted analyses, and post-discharge follow-up is needed to determine whether the observed psychological differences persist after accounting for baseline differences between patients.
